# Supplementary material for: The coverage of maternal continuum-of-care and associated factors in the Lao People’s Democratic Republic: A population-based cross-sectional study
Source: PLoS One. 2026 Mar 25;21(3):e0345660. doi: 10.1371/journal.pone.0345660 (PMC13016336; doi:10.1371/journal.pone.0345660)
Supplement: S1 Table — (DOCX) [file pone.0345660.s001.docx]

**S1 Table. Definition and categorization of obstetric variables.**

| **Variable** | **Definition/categories** |
| --- | --- |
| Number of children | 1. One child 2. ≥2 children |
| Number of ANC visits | 1. 0–3 2. 4–7 3. ≥8 |
| Gestational age at the first ANC visit | 1. ≤12 weeks 2. >12 weeks |
| ANC provider | 1. Doctor 2. Nurse/midwife 3. Other (traditional birth attendant, village health volunteer, other) |
| Mode of delivery | 1. Vaginal delivery 2. Cesarean section |
| Delivery assistant | 1. Doctor 2. Nurse/midwife 3. Other (traditional birth attendant, village health volunteer, relative/friend, other) |
| Place of delivery | 1. Hospital 2. Health center 3. Private clinic |
| Size of the last child at birth | 1. Large 2. Average 3. Small |
| Sex of the last child | 1. Male 2. Female |
| Maternal health check before discharge | 1. Yes 2. No |
| PNC visit after delivery within six weeks | 1. Yes 2. No |

ANC, antenatal care; PNC, postnatal care.
