# Supplementary material for: The coverage of maternal continuum-of-care and associated factors in the Lao People’s Democratic Republic: A population-based cross-sectional study
Source: PLoS One. 2026 Mar 25;21(3):e0345660. doi: 10.1371/journal.pone.0345660 (PMC13016336; doi:10.1371/journal.pone.0345660)
Supplement: S2 Table — (DOCX) [file pone.0345660.s002.docx]

**S2 Table. Comparison of characteristics between included and excluded women.**

| **Variable** | **Total** | **Included women** | **Excluded women** | ***P*-value^c^** |
| --- | --- | --- | --- | --- |
|  | **(n = 3,448)** | **(n = 2,612)** | **(n = 836)** |  |
|  | n (%) | n (%) | n (%) |  |
| **Age (years old)** | | | |  |
| 15–19 | 492 (14.3) | 362 (13.9) | 130 (15.6) | **<0.001** |
| 20–29 | 1,897 (55.0) | 1,447 (55.4) | 450 (53.8) |  |
| 30–39 | 960 (27.8) | 745 (28.5) | 215 (25.7) |  |
| 40–49 | 99 (2.9) | 58 (2.2) | 41 (4.9) |  |
| **Educational level** | | | |  |
| None/elementary | 1,447 (41.9) | 869 (33.3) | 578 (69.1) | **<0.001** |
| Secondary | 1,495 (43.4) | 1,250 (47.9) | 245 (29.3) |  |
| Post-secondary/tertiary | 506 (14.7) | 493 (18.8) | 13 (1.6) |  |
| **Ethnicity** | | | |  |
| Lao-Tai | 1,740 (50.5) | 1,522 (58.3) | 218 (26.1) | **<0.001** |
| Mon-Khmer | 997 (28.9) | 643 (24.6) | 354 (42.3) |  |
| Hmong-Mien | 549 (15.9) | 355 (13.6) | 194 (23.2) |  |
| Chinese-Tibetan | 140 (4.1) | 73 (2.8) | 67 (8.0) |  |
| Others | 22 (0.6) | 19 (0.7) | 3 (0.4) |  |
| **Residential region** | | | |  |
| Northern | 1,170 (33.9) | 820 (31.4) | 350 (41.9) | **<0.001** |
| Central | 1,656 (48.0) | 1,333 (51.0) | 323 (38.6) |  |
| Southern | 622 (18.1) | 459 (17.6) | 163 (19.5) |  |
| **Living area** | | | |  |
| Urban | 889 (25.8) | 814 (31.2) | 75 (9.0) | **<0.001** |
| Rural | 2,559 (74.2) | 1,798 (68.8) | 761 (91.0) |  |
| **Wealth index quintile** | | | |  |
| Low/middle | 2,378 (69.0) | 1,590 (60.9) | 788 (94.3) | **<0.001** |
| High | 1070 (31.0) | 1022 (39.1) | 48 (5.7) |  |
| **Marital status** | | | |  |
| Married | 3,346 (97.0) | 2,538 (97.2) | 808 (96.7) | 0.448 |
| Divorced/widowed/never married | 102 (3.0) | 74 (2.8) | 28 (3.3) |  |
| **Age at first marriage (years old)** | | | |  |
| ≤ 19 | 2,005 (58.2) | 1,375 (52.6) | 630 (75.4) | **<0.001** |
| 20–49 | 1443 (41.8) | 1,237 (47.4) | 206 (24.6) |  |
| **Health insurance coverage** | | | |  |
| No | 2,185 (63.4) | 1,585 (60.7) | 600 (71.8) | **<0.001** |
| Yes | 1,263 (36.6) | 1,027 (39.3) | 236 (28.2) |  |
| **Number of ANC visit (n = 3,446)** | | | |  |
| 0–3 | 969 (28.1) | 442 (16.9) | 527 (63.2) | **<0.001** |
| 4–7 | 1765 (51.2) | 1507 (57.7) | 258 (30.9) |  |
| 8 times or more | 712 (20.7) | 663 (25.4) | 49 (5.9) |  |
| **Delivery assistant (n = 3,446)** | | | |  |
| Doctor | 2,207 (64.0) | 2,100 (80.4) | 107 (12.8) | **<0.001** |
| Nurse/midwife | 546 (15.8) | 476 (18.2) | 70 (8.4) |  |
| Other^a^ | 667 (19.4) | 36 (1.4) | 631 (75.7) |  |
| No assistant | 26 (0.8) | 0 (0.0) | 26 (3.1) |  |
| **Place of delivery (n = 3,438)** | | | |  |
| Home | 736 (21.4) | 0 (0.0) | 736 (89.1) | **<0.001** |
| Health facilities^b^ | 2,702 (78.6) | 2,612 (100.0) | 90 (10.9) |  |
| **PNC visit after discharge within six weeks postpartum (n = 2,700)** | | | |  |
| Yes | 101 (3.7) | 98 (3.8) | 3 (3.4) | **<0.001** |
| No | 2,599 (96.3) | 2,514 (96.2) | 85 (96.6) |  |

ANC, antenatal care; PNC, postnatal care.

^a^Other includes traditional birth attendants and village health volunteers.

^b^Health facilities include hospitals, health centers, and private clinics.

^c^Chi-square or Fisher’s exact tests were used.
