# Supplementary material for: The coverage of maternal continuum-of-care and associated factors in the Lao People’s Democratic Republic: A population-based cross-sectional study
Source: PLoS One. 2026 Mar 25;21(3):e0345660. doi: 10.1371/journal.pone.0345660 (PMC13016336; doi:10.1371/journal.pone.0345660)
Supplement: Attachment — Submitted filename: Reviewer Attachment_PONE-D-25-57622_Kyaw Zayar Aung .docx [file pone.0345660.s003.docx]

| Line # | Finding | Suggestions |
| --- | --- | --- |
| 134 | “never married” | In many Asian cultural contexts, women typically pursue pregnancy within marriage. Therefore, the definition of the variable “never married” requires clarification. If “never married” refers to women who have never been in a marital union, they would logically fall under the exclusion criteria. Alternatively, if “never married” includes single mothers or women who became pregnant outside of marriage, this should be explicitly stated.  I would like to recommend that the authors clarify the operational definition of “never married” in the study. |
| 229 | ANC Provider “Other” and Place of Delivery | In Line 150, the “Other” category for ANC providers includes traditional birth attendants, village health volunteers, and relatives. Women who choose providers in this category are often more likely to give birth at home or in locations outside formal health facilities.  In Table 2, the authors report that 33 (1.3%) of women delivered with providers categorized as “Other.” However, there appears to be no corresponding category defined in Lines 153–154 or clearly reflected in the “Place of Delivery” in Table 2.  I recommend that the authors clarify the definition of the “Other” ANC provider category and verify these data across the manuscript. |
| 275-277  298 | Family permission  “Women who received postpartum checks..” | I strongly support the authors’ point, which aligns with the socio-cultural context in many Asian settings. In these societies, decision-making regarding antenatal and postnatal care is often influenced by husbands and elder family members.  Therefore, I would like to recommend that the authors consider adding wording that highlights the importance of engaging husbands and senior family members in promoting ANC and PNC utilization. |
| 300-301 | Such information and counseling  could have facilitated subsequent utilization of PNC services. | From a practical implementation perspective, I would like to suggest that the authors consider adding the local language(s) in this point to be better understanding, to enhance comprehension, ensure cultural relevance, and improve the effectiveness of PNC promotion efforts. |
| 310-322 | Different strategy | The authors correctly highlight key elements such as improving knowledge and implementing culturally appropriate strategies. Previous studies have demonstrated that context-specific approaches, including home visits in geographically challenging or mountainous regions, can be particularly effective. Additionally, the involvement of ethnic community leaders has been shown to play an important role in promoting ANC and PNC uptake within their communities.  From a programmatic and practical standpoint, I would like to recommend that the authors consider elaborating on geographically and culturally tailored interventions such as home-based outreach and collaboration with ethnic community leaders to strengthen the applicability of their recommendations. |
