# Supplementary material for: The coverage of maternal continuum-of-care and associated factors in the Lao People’s Democratic Republic: A population-based cross-sectional study
Source: PLoS One. 2026 Mar 25;21(3):e0345660. doi: 10.1371/journal.pone.0345660 (PMC13016336; doi:10.1371/journal.pone.0345660)
Supplement: Attachment — Submitted filename: Comments to authors.docx [file pone.0345660.s004.docx]

**Comments to authors**

Regarding the outcome measures, line number 162-167 say “the continuum-of-care was defined as receiving ANC at least four times, delivery attended by an SBA; including doctors, midwives, and nurses, and at least one PNC visit after discharge from a health facility within six weeks postpartum. Participants who received all three components were considered to have completed the continuum-of-care. Due to the limited number of women who received PNC, **having at least one PNC visit within six weeks after delivery was deemed sufficient to meet the PNC criterion.**”

So, among 2617 study participants, 2170 got more than 4 ANC visits, 2147 got SBA delivery but only 86 got PNC. Given that nearly all women delivered in health facilities (n = 2,612), including 252 cesarean sections, it is unclear whether women who had SBA-assisted institutional deliveries did not receive any postnatal assessment, including within 24 hours of delivery. Postnatal care was operationalized as a separate maternal health check occurring after discharge from the delivery facility. This strongly suggests that routine postpartum checks before discharge were not classified as PNC.

This results low prevalence of complete continuum of care (3.3%) largely due to the restricted operational definition of postnatal care rather than a true absence of services, which may affect the outcome estimates and may create a highly imbalanced outcome distribution which could affect the precision and stability of multivariable regression estimates.

As a result, the low outcome prevalence (3.3%) leads to a highly imbalanced outcome distribution and a relatively small number of events despite the large study population. Under these conditions, conventional logistic regression would be prone to small-sample bias, overfitting of predictors, and an increased risk of type I error. Reducing the number of predictors or applying penalized logistic regression methods is therefore recommended to minimize overfitting and prevent inflation of odds ratio estimates.
