# Supplementary material for: The coverage of maternal continuum-of-care and associated factors in the Lao People’s Democratic Republic: A population-based cross-sectional study
Source: PLoS One. 2026 Mar 25;21(3):e0345660. doi: 10.1371/journal.pone.0345660 (PMC13016336; doi:10.1371/journal.pone.0345660)
Supplement: Attachment — Submitted filename: Response to reviewers.docx [file pone.0345660.s005.docx]

**Response to the editor’s and reviewers’ comments**

We would like to thank the editor and reviewers for reviewing our manuscript. We have revised the manuscript according to your comments, which were very helpful. The revised manuscript has been proofread by a native English speaker. The revisions have been completed and our responses are as follows.

**Journal requirements:**

1. Please ensure that your manuscript meets PLOS ONE's style requirements, including those for file naming. The PLOS ONE style templates can be found at

https://journals.plos.org/plosone/s/file?id=wjVg/PLOSOne_formatting_sample_main_body.pdf and

https://journals.plos.org/plosone/s/file?id=ba62/PLOSOne_formatting_sample_title_authors_affiliations.pdf

Author response:

We have carefully revised the manuscript to ensure that it meets PLOS ONE’s style requirements.

2. Thank you for uploading your study's underlying data set. Unfortunately, the repository you have noted in your Data Availability statement does not qualify as an acceptable data repository according to PLOS's standards.

At this time, please upload the minimal data set necessary to replicate your study's findings to a stable, public repository (such as figshare or Dryad) and provide us with the relevant URLs, DOIs, or accession numbers that may be used to access these data. For a list of recommended repositories and additional information on PLOS standards for data deposition, please see https://journals.plos.org/plosone/s/recommended-repositories.

Author response:

Thank you for your guidance regarding the Data Availability requirements.

In response to your comment, we have uploaded the minimal data set necessary to replicate the findings of our study to a stable, public repository in accordance with PLOS ONE’s data sharing standards. Specifically, the derived and fully anonymized dataset used for the analyses has been deposited in figshare.

Repository: figshare
DOI: https://doi.org/10.6084/m9.figshare.31145893

This dataset represents the minimal data set required to reproduce the results reported in the manuscript and does not contain any personally identifiable information. The data were derived from the Lao Social Indicator Survey III (LSIS III), conducted as part of UNICEF’s Multiple Indicator Cluster Surveys (MICS).

Lines 217-218: We have revised the Data Availability statement in the Methods section of the manuscript to reflect this update. The revised statement now indicates that the minimal dataset is publicly available via figshare, while the original LSIS III data remain accessible from the UNICEF MICS repository upon user registration and approval, in accordance with their data use policy. We hope that these revisions fully address the journal’s requirements.

3. Your ethics statement should only appear in the Methods section of your manuscript. If your ethics statement is written in any section besides the Methods, please move it to the Methods section and delete it from any other section. Please ensure that your ethics statement is included in your manuscript, as the ethics statement entered into the online submission form will not be published alongside your manuscript.

Author response:

We have carefully checked the manuscript and confirmed that the ethics statement appears only once, under the “Ethical considerations” subsection within the Methods section. There is no duplicate ethics statement in any other section of the manuscript.

4. If the reviewer comments include a recommendation to cite specific previously published works, please review and evaluate these publications to determine whether they are relevant and should be cited. There is no requirement to cite these works unless the editor has indicated otherwise.

Author response:

There was no comment from the reviewers that includes a recommendation to cite specific previously published works.

**Reviewer #1:** The study is on cross-sectional study that focuses on maternal continuum-of-care coverage in Lao PDR based on nationally representative data. Extremely low completion rates (3.3) and related factors are the result of the authors. Although the subject is significant, and the data are strong, several methodological and interrogatory issues should be resolved.
1-1. It is very large (30.7% of the participants) that 1,156 women were not included because of missing data, and this is not sufficiently discussed. The authors admit that these were mostly home deliveries; however, they do not examine the nature of the disqualified women and leave it unnoticed how this impacts the results. This highly restricts the ability to generalize, because the research, in essence, only analyzes facility-based births and concludes on coverage in the nation. Perform sensitivity analysis of the features of included and excluded women. Make it clear in the abstract and other conclusions that findings are relevant to facility-based births.
Author response:

Thank you for this important and constructive comment. We applied sampling weights for all analyses and Fig 1 has been revised. Of 3,448 women (weighted) had given birth in the past 2 years, 2,612 (weighted) were included and 836 (weighted) were excluded.

We conducted a sensitivity analysis comparing women included in the final analysis with those excluded due to missing data on key outcome variables. The results of this analysis are presented in a new table (Suppl Table). Statistically significant differences were observed between the two groups across most socio-demographic and obstetric characteristics. Excluded women were more likely to have no or an elementary education, belong to ethnic minority groups as Mon-Khmer, reside in rural areas, be classified as low/middle wealth index, be married at age 19 years or younger, and have no health insurance. Nearly all excluded women delivered at home.

Lines 387-398: In response, we have revised the Limitations section to clearly state that the findings primarily reflect patterns of maternal healthcare utilization among women who delivered in health facilities and may not be generalizable to home deliveries as follows, “This study has some limitations. First, 836 of the 3,448 women who had given live birth within two years before the survey were excluded because of missing data on key variables. The excluded women differed systematically from those included, being predominantly home births and more likely to have low socioeconomic status, including having lower educational attainment, belonging to ethnic minority groups, residing in rural areas, lower/middle wealth index, being married at age 19 years or younger, and having no health insurance (Suppl Table). As these characteristics are associated with lower utilization of maternal healthcare services, this exclusion may have resulted in an overestimation of the completion of the continuum-of-care. Consequently, the findings primarily reflect maternal healthcare utilization among facility-based births and may not be fully generalizable to women who had home delivery. For contextual reference, if all excluded women were classified as not completing the continuum-of-care, the overall completion rate would be 2.5% (86/3,448).”

1-2. The definition applied (≥4 ANC visits, SBA delivery, ≥1 PNC visit) is not as rigorous as the WHO recommendations (8 ANC visits, PNC at specific intervals). Although the authors explain this by the low PNC uptake, a low threshold can inflate actual continuum completion and restrict the comparison with other research. Additional analysis with WHO-suggested thresholds or limitations should be mentioned, or limitations should be discussed more clearly.
Author response:

Lines 181-183: Thank you for the comment. We acknowledge that the definition of maternal continuum-of-care used in this study is less strict than current WHO recommendations, which include at least eight antenatal contacts and PNC at specific time intervals. Due to limitations in available LSIS III variables and the extremely low uptake of PNC, applying WHO-recommended thresholds was not feasible in this study. Regarding the number of ANC visits, we have added a sentence to the Methods section as follows, “Although the WHO recommends ≥ 8 ANC contacts, this was not fully implemented in Lao PDR during the LSIS III period, and the national strategy still uses ≥ 4 ANC visits as an indicator.”

Lines 398-402: We have revised the Limitations section to clearly state that the use of lower thresholds may overestimate continuum-of-care completion and restrict comparability with studies using WHO-recommended definitions as follows, “Second, the definition of maternal continuum-of-care in this study is less stringent than current WHO recommendations regarding the number of ANC visits and was necessitated by data constraints and very low PNC uptake, which may limit comparability with other studies. Completion rate of the continuum-of-care when using at least ANC 8 visits was 1.7% (45/2,612).”

1-3. There is a total of 11 variables and only 86 outcomes in the multivariate model, which can overfit (ratio of 8 events per variable). There is no multicollinearity assessment discussion. Not describe the variable selection process. Reduce adjusted selection of variables, report model diagnostics, and think about a more parsimonious model.
Author response:

We thank the reviewer for raising important concerns regarding potential overfitting and model specification.

We acknowledge that the multivariable model includes 11 variables with 86 outcome events, which may raise concerns about the events-per-variable ratio. However, all variables were selected a priori based on established literature and theoretical relevance to control for potential confounding, rather than through data-driven selection procedures.

To address concerns about model stability, we assessed multicollinearity among all covariates using VIFs. All VIF values (1.009-1.743) were below commonly accepted thresholds (< 5.0), indicating no evidence of problematic multicollinearity. In addition, we conducted sensitivity analyses using alternative, more parsimonious models including only key covariates. The direction and magnitude of the main effect estimates were consistent with those of the primary model, supporting the robustness of our findings.

Lines 199-205: We have added sentences in the Methods section as follows, “Selection of variables for the multivariable model was determined a priori based on prior literature and theoretical relevance, with the aim of adequately controlling for potential confounding factors. To assess potential multicollinearity among covariates, variance inflation factors (VIFs) were calculated for all variables included in the multivariable model. All VIF values were below commonly accepted thresholds, indicating no evidence of problematic multicollinearity. Model diagnostics were performed to evaluate the adequacy of the final model and model fit was assessed using the Hosmer–Lemeshow goodness-of-fit test.”

Lines 282-287: We have added sentences in the Results section as follows, “Sensitivity analyses were conducted to assess the robustness of the multivariable model. Alternative models, including only key covariates selected based on theoretical importance, were examined. The direction and magnitude of the main effect estimates were consistent with those observed in the primary model, indicating that the results were not materially affected by the number of covariates included.”

We believe these additional analyses and clarifications adequately address the reviewer’s concerns regarding overfitting and model robustness.

1-4. The terms associated with and factors, throughout the manuscript, imply causation despite the cross-sectional design. Indicatively, such as the fact that having the last child as a male was linked to completion indicates that male child sex implies care-seeking, yet one cannot tell whether it was the timing. Be more careful in language, and focus on correlational results.
Author response:

Thank you for this important comment. We agree that causal language should be avoided considering the cross-sectional design. We have revised the manuscript throughout to replace causal wording with correlational language.

1-5. PNC variable of interest includes "at least one PNC visit in the six weeks following discharge," but does not differentiate between facility-based PNC and home-based PNC, and the timing of the visit(s). This curtails the meaning of the "continuum."
Author response:

We thank the reviewer for this thoughtful comment regarding the definition of PNC and its implications for the concept of the continuum of care. In this study, PNC was defined as having at least one PNC visit within six weeks following discharge from a health facility. This definition was adopted because facility-based maternal health checks before discharge are routinely provided following facility delivery in Lao PDR and therefore do not meaningfully differentiate PNC utilization after childbirth. Our focus was thus on post-discharge care, which reflects continued engagement with maternal health services beyond delivery. We acknowledge that the available LSIS III data do not allow further differentiation between facility-based and home-based PNC, nor do they capture the precise timing of PNC visits within the six-week period. We agree that this limits the granularity with which the continuum-of-care can be characterized. We have clarified this rationale in the Methods section and explicitly acknowledged this limitation in the Discussion. Despite these constraints, we believe that the post-discharge PNC indicator remains a meaningful measure of continuity of maternal health care within the context of the available data. We have revised the manuscript to more clearly describe the scope and limitations of this definition.

Lines 176-180: We have revised the sentence in the Methods section as follows, “the continuum-of-care was defined as receiving ANC at least four times, delivery attended by an SBA; including doctors, midwives, and nurses, and at least one PNC visit after discharge from a health facility within six weeks postpartum, as routine facility-based checks prior to discharge are nearly universal and do not capture continued care-seeking after delivery.”

Lines 402-408: We have added a sentence in the Discussion section as follows, “Third, the definition of maternal continuum-of-care in this study included post-discharge PNC but not maternal check before discharge, because routine facility-based checks before discharge were considered nearly universal. However, 19.3% of the mothers in this study answered that they did not receive a maternal health check. Furthermore, the LSIS III data does not allow differentiation of PNC by location or precise timing, which limits the ability to fully characterize the continuum-of-care.”

1-6. The theme of a gender preference (lines 284-285) should be discussed more subtly. The male-child care completion relationship may represent various processes other than the cultural preference for sons.
Author response:

Lines 316-327: Thank you for this important comment. We have revised the Discussion section to address this concern by adopting a more nuanced interpretation of the observed association as follows, “Gender-related disparities in health service utilization have been reported in settings with son preference [40]. Sons are often viewed as important for family continuity and as a source of labor in rural settings, and, in contexts where formal social security systems are limited, they may be perceived as providing future support in old age [40]. In Lao PDR, cultural norms and gender roles may still influence family decision-making regarding pregnancy and childbirth care [41]. In addition, biological and clinical factors may also contribute to this association. Previous studies have reported a higher incidence of stillbirth, neonatal morbidity and mortality, preterm delivery, and congenital anomalies among male fetuses compared with females [32, 42-44], which may increase perceived pregnancy risk and influence care-seeking behaviors. Taken together, these findings suggest that the observed association likely reflects a combination of social, cultural, and biological factors, and causal interpretations cannot be made given the cross-sectional design of this study.”

1-7. No data were given on the handling of missing data on covariates in regression models. Did it exclude cases that had any covariate data that were missing?
Author response:

Lines 196-197: Thank you for this comment. We apologize for the lack of clarity in the original manuscript. There were no missing data for any covariates included in the regression models; therefore, all eligible women were included in the analyses. We have clarified this point in the Methods section as follows, “There were no missing data for any covariates included in the regression analyses.”

1-8. The variable of health insurance is an unreasonable combination of two schemes. These can include other levels of coverage and, therefore, may influence the use of care differently.
Author response:

Thank you for this important comment. First, we apologize that private insurance was also included in “health insurance coverage.” We acknowledge that the national health insurance (NHI), social health insurance (SHI), and private insurance schemes in Lao PDR differ in their target populations and levels of coverage, and may therefore influence maternity care utilization differently. In our dataset, however, the number of women who completed the continuum of maternity care within the SHI (n=5) and private insurance (n=6) groups was very small, which precluded stable estimation of scheme-specific effects in multivariable regression models. For this reason, we combined NHI, SHI, and private insurance into a single “insured” category. We have clarified this rationale in the Methods section and added this point as a limitation in the Discussion section, noting that potential differences across insurance schemes could not be examined in the present study.

Lines 144-148: We have revised the Methods section as follows, “Health insurance coverage was coded as “yes” if participants were covered by either the national health insurance scheme, the social health insurance scheme, or private insurance, and “no” if they had no coverage. The three schemes were combined because all provide financial protection for maternal and child health services, and analysis was not feasible due to the limited sample size within individual schemes.”

Lines 408-412: We have added sentences in the Discussion section as follows, “Fourth, three health insurance schemes included in this study differ in their coverage and target populations. Because of the small number of women completing the continuum-of-care within each scheme, we were unable to examine scheme-specific associations. As a result, differences in the effects of individual insurance schemes on maternity care utilization may have been obscured.”

1-9. Table 3: It might be worthwhile to drop non-significant variables from the adjusted model or stepwise.

Author response:

Thank you for this suggestion. We agree that reducing the number of covariates or using stepwise selection can be useful to improve model parsimony, particularly when the number of outcome events is limited. In the present study, however, the adjusted model was specified a priori to control for potential confounding based on established literature and theoretical considerations, rather than statistical significance alone. For this reason, we did not remove non-significant covariates from the primary adjusted model or apply stepwise procedures. As noted in our response to the previous comment No. 3, we conducted sensitivity analyses using more parsimonious models that included only key covariates. The direction and magnitude of the main effect estimates were consistent with those of the primary model, indicating that the results shown in Table 3 are robust and not materially influenced by the inclusion of non-significant variables.

1-10. The SBA coverage of 98.6 percent does not appear to be in line with 827 home deliveries among excluded women.

Author response:

Thank you for this important comment. We understand the concern regarding the apparent inconsistency between the high SBA coverage (98.6%) and the large number of home deliveries among excluded women. We would like to clarify that the SBA coverage of 98.6% was calculated only among the analytic sample of 2,612 women, all of whom reported facility-based delivery and had complete information on ANC, delivery assistant, and PNC. Women who delivered at home were excluded from the main analysis because they did not respond to key outcome variables, including the number of ANC and PNC visits. As a result, the reported SBA coverage reflects utilization among women delivering in health facilities and does not represent SBA coverage among all women who gave birth in the two years preceding the survey. To address this issue and improve transparency, we have added S1 Table ( comparing the characteristics of included and excluded women, including place of delivery and birth assistant.

1-11. Line 222: Expand upon, all 2612 women delivered at the health facility, since home deliveries have already been mentioned above.

Author response

Lines 231-235: We have added explanation that all subjects of this study were women who had childbirth at health facilities because all women who had home delivery were excluded in the Methods section as follows, “Women who delivered at home (n = 736) were excluded from the analytical sample; therefore, all 2,612 women included in the analysis had facility-based deliveries. Characteristics of included and excluded women, including those with home deliveries, were compared and are presented in S1 Table.”

1-12. The discussion may be made shorter. Certain paragraphs represent result information (e.g., lines 258-266).
Author response

Thank you for this helpful comment. We agree that parts of the Discussion section repeated results, particularly in lines 258–266 of the original manuscript. We have revised the Discussion to improve conciseness by removing descriptive result information and focusing more on interpretation and implications. Specifically, numerical results and detailed coverage estimates were deleted or relocated to the Results section, and the corresponding Discussion paragraphs were streamlined accordingly. While the total length of the Discussion did not substantially decrease due to the incorporation of reviewers’ suggestions, we have ensured that result repetition was minimized and that added text focused on interpretation, contextualization, and practical implications.

1-13. Missing affiliation number 4 - The sequence of check numbering.

Author response

We are sorry to make a mistake of the affiliation number 3 and 4. We have revised correctly.

1-14. Line 269: postpartum hemorrhage is the leading cause - must be cited.

Author response:

We have added a reference for this sentence (reference No. 34).

Say L, Chou D, Gemmill A, Tuncalp O, Moller AB, Daniels J, et al. Global causes of maternal death: a WHO systematic analysis. Lancet Glob Health. 2014;2(6):e323-33. Epub 2014/08/12. doi: 10.1016/S2214-109X(14)70227-X. PubMed PMID: 25103301.

1-15. Surely, Tables 1 and 2 are worth combining or shifting to the annexes.
Author response:

Thank you for this suggestion. Tables 1 and 2 present different types of baseline characteristics: Table 1 summarizes socio-demographic factors, whereas Table 2 focuses on obstetric characteristics. We chose to present these tables separately to enhance clarity and readability. Combining the two tables would result in a substantially longer and more complex table, which may reduce readability for readers. For this reason, we believe that presenting these variables in two separate tables is more appropriate. However, we are willing to revise the table structure if the editor considers consolidation or relocation preferable.

1-16. Abstract: Indicate that this analysis is limited to the facility-based births.

Author response:

Lines 28-29: We have revised the abstract and added as follows, “In the selection process of the study subjects, all women who had home delivery were excluded due to missing data on the three components of the continuum-of-care. Of the 2,612 women (with facility-based delivery), 83.1% received…”

**Reviewer #2**: This manuscript addresses an important public health issue: the very low completion of the maternal continuum-of-care (CoC) in Lao PDR. The paper is clearly structured, uses nationally representative data (LSIS III), and applies appropriate statistical methods. The major strengths are the use of high-quality population survey data and the identification of socio-cultural determinants—including ethnicity, wealth, and gender norms.
Overall, the study is valuable and relevant, but revisions would improve its rigor and clarity.

2-1. Sample selection and potential bias
A large proportion of eligible women (approximately 31%) were excluded due to missing ANC/PNC data, primarily among those who delivered at home. This exclusion likely introduces systematic bias and may underestimate true CoC coverage. Can you describe the characteristics of excluded vs. included women (if available), the reason for missingness in LSIS III and how this exclusion may influence the results and their generalizability?
Author response:

Thank you for this important comment. We have made S1 Table (Supporting information) to show the characteristics of included women (study subjects) and excluded women. Excluded women differed systematically from those included in the analysis. Excluded women were more likely to have no or an elementary education, belong to ethnic minority groups, such as the Mon-Khmer, reside in rural areas, be classified as having a low/middle wealth index, be married at the age of 19 years or younger, and have no health insurance.

The reason for missing data in LSIS III was related to home deliveries, as information on ANC and PNC, key components of the continuum of care, was incomplete among women who gave birth at home. We have revised the Methods, Results, and Discussion sections. Please refer to our response to Comment No. 1-1 (from Reviewer 1).

2-2. Definition of continuum-of-care
The study uses ≥4 ANC visits as the ANC criterion, whereas current WHO guidance recommends ≥8 contacts. Please provide a justification why you select the 4-visit threshold.
Author response:

Thank you for this comment. Although the WHO recommended ≥ 8 ANC contacts in 2016, this has not yet been fully implemented in Laos during the LSIS III period. The national strategy still uses ≥ 4 ANC visits as the standard, so we used this threshold as our ANC criterion. Please read our response to Comment No. 1-2 (from Reviewer 1).

2-3. Multivariate Model Specification Needs More Explanation
The selection of variables for the multivariate model is not fully transparent. Please clarify which criteria us used (e.g., all variables with P<0.2 in univariate analysis were included), or whether theoretical justification was used. It is important to consider reporting model diagnostics (multicollinearity, goodness-of-fit). In results section, it is good to use " how many times higher or lower" instead of jargon (aOR).
Author response:

We thank the reviewer for raising important concerns regarding potential overfitting and model specification. We selected variables for the multivariate model based on established literature and theoretical relevance to control for potential confounding, rather than through data-driven selection procedures such as univariate screening. To address concerns about model stability, we assessed multicollinearity among all covariates using variance inflation factors (VIFs). All VIF values (range: 1.009–1.743) were below commonly accepted thresholds (<5.0), indicating no evidence of problematic multicollinearity. In addition, model fit was assessed using the Hosmer–Lemeshow goodness-of-fit test, which indicated no evidence of lack of fit (P = 0.216). To further address concerns regarding overfitting, we conducted sensitivity analyses using alternative, more parsimonious models that included only key covariates selected based on theoretical importance. The direction and magnitude of the main effect estimates were consistent with those of the primary model, supporting the robustness of our findings.

Lines 199-205: We have added sentences in the Methods section as follows, “Selection of variables for the multivariable model was determined a priori based on prior literature and theoretical relevance, with the aim of adequately controlling for potential confounding factors. To assess potential multicollinearity among covariates, variance inflation factors (VIFs) were calculated for all variables included in the multivariable model. All VIF values were below commonly accepted thresholds, indicating no evidence of problematic multicollinearity. Model diagnostics were performed to evaluate the adequacy of the final model and model fit was assessed using the Hosmer–Lemeshow goodness-of-fit test.”

Lines 275-282: To improve interpretability, we have revised the Results section to describe adjusted odds ratios using plain language (e.g., “X times higher/lower odds”), while retaining AOR values and confidence intervals for statistical clarity as follows, “Multivariate logistic regression analysis showed that being classified as having a high wealth index had approximately two times higher odds of completing the maternal continuum-of-care (adjusted odds ratio [AOR] = 2.19, 95% CI: 1.19–4.04, P = 0.012). Similarly, having the last child as male was associated with 1.65 times higher odds (AOR = 1.65, 95% CI: 1.04–2.61, P = 0.032), and receiving a maternal health check before discharge were associated with completion of the maternal continuum-of-care (AOR = 2.40, 95% CI: 1.06–5.41, P = 0.035). In contrast, Hmong-Mien women had statistically lower odds of completing the continuum-of-care than Lao-Tai women (AOR = 0.24, 95% CI: 0.06–0.91, P = 0.036).”

Minor Issues
2-4. Correct typographical errors in tables (e.g., % of Non/ Elementary Education Level should be 33.3%. in Table 1).
Author response:

Thank you for the comment. We have revised the error (33.3% is correct).

2-5. Ensure Figure 1 is clearly labeled, legible, and matches the text description.
Author response: Figure 1 has been revised to improve clarity and consistency with the text. The figure title and legend were updated to clarify the use of weighted and unweighted numbers.

2-6. Table 2 footnote: “Other includes traditional birth attendants and village health volunteers”—is needed to be consistent with explanation in Line no. 150.
Author response:

In the original questionnaire of LSIS III, choices for birth attendants included doctors, nurses/midwives, traditional birth attendants, village health volunteers, and friends/relatives. Of the 2,612 women included in the study, no one had friends/relatives as birth assistants. However, we have added S1 Table, which showed women were excluded from the study and some of them had friends/relatives as birth assistant. Therefore, we have revised “relatives” to “friends/relatives” in the Methods section (Line 157).

2-7. Standardize terminology (e.g., “upper/high wealth index” in Line no. 338).

Author response:

We apologize for the inconsistent use of terminology in the manuscript. We carefully reviewed and revised the manuscript to ensure that terminology is used consistently and in a standardized manner throughout.

**Reviewer #3**: Reviewer Comments on “The coverage of maternal continuum-of-care and associated factors in the Lao People’s Democratic Republic: A population-based cross-sectional study.”
Thank you for the opportunity to review this manuscript. The study found that maternal continuum-of-care coverage in Lao PDR remains very low, with significant gaps across antenatal, delivery, and most remarkably postnatal services, and is influenced by socioeconomic, demographic, and geographic factors. Using secondary data from the Lao Social Indicator Survey III (LSIS III, 2023), researchers conducted a population-based cross-sectional analysis to assess the extent of maternal continuum-of-care (CoC) - defined as receiving antenatal care (ANC), skilled birth attendance (SBA), and postnatal care (PNC). The findings revealed that only a small proportion of women completed the full continuum, with drop-offs most pronounced between SBA and PNC.
3-1. Abstract: “Of the 2,612 women, 83.1% had at least four ANC visits, 98.6% had SBA delivery, 3.8% had at least one PNC visit, and 3.3% completed the maternal continuum-of-care.” There may need to be added conjunctions like “of which 98.6% had SBA delivery, of which 3.8% had at least one PNC visit, …” or something like this, to make visible that these proportions were continuous or cascaded.
Author response:

We agree with the reviewer that clarity regarding the continuity of care is essential. To improve interpretability, we revised the Abstract and the Results section to present each stage of the maternal continuum-of-care as the proportion of the total study population that reached that stage, thereby making the sequential nature of care explicit while maintaining a consistent denominator.

Lines 29-31: We have revised the sentence in Abstract as follows, “Of the 2,612 women (with facility-based delivery), 83.1% received ≥ 4 ANC visits, 82.2% received both ≥ 4 ANC visits and SBA delivery, and 3.3% completed the maternal continuum-of-care.”

Lines 225-231: We have revised the Results section as follows, “Among these women, 83.1% (n = 2,170) received at least four ANC visits, 98.6% (n = 2,576) had delivery attended by an SBA, and 3.8% (n = 98) received at least one PNC visit after discharge. Overall, 82.2% (n = 2,147) received both ≥ 4 ANC visits and SBA-attended delivery. Only 3.3% (n = 86) completed the maternal continuum-of-care by receiving ≥ 4 ANC visits, SBA-attended delivery, and at least one PNC visit after discharge. The coverage declined markedly across the continuum, with the largest drop observed between SBA-attended delivery and PNC.”

3-2. Study Design: Line 105 - Sample size (15-49 women) in LSIS III was 22,126. Is there any explanation why some were excluded from 22,512? It would be better to describe it shortly here.
Author response:

During the LSIS III survey, 22,512 women aged 15–49 years were identified in 20,325 households. Of these, 2,487 women did not participate in the interview, resulting in a final sample of 22,126 women included in the LSIS III dataset. As this study is a secondary analysis of the LSIS III data, detailed reasons for non-participation were not available in the LSIS III report.

3-3. Line 112 - The proportion of deliveries within two years looks quite small at about 17%. Any comparison within the country or regions in recent years stated in LSIS III? It would be better to describe the other RH indicators.
Author response:

Thank you for this comment.
According to LSIS III, among 22,512 women aged 15–49 years (unweighted), 3,448 women reported having had a live birth in the two years preceding the survey, corresponding to 15.3% of women of reproductive age. This proportion is consistent with the national reproductive health context in Lao PDR as reported in LSIS III. While coverage of ≥ 4 ANC visits and SBA delivery is relatively high at the national level, PNC coverage remains substantially lower. These patterns suggest that the observed proportion of recent deliveries reflects demographic trends rather than under-representation in the survey. This proportion is also broadly consistent with population-based estimates from international sources, which suggest that approximately 15–16% of women aged 15–49 years would be expected to have given birth within a two-year period.

3-4. Line 112 - In this study, “Women who had childbirth within the 2 years before the interview (N= 3,768)”, I have found that the Number of women with a live birth in the last 2 years was 3448 in the Tables of ANC and PNC. Stillbirths included in your study with 3768 women? If so, could you please explain more? Usually, CoC completion is with live births, but in some cases, SB were included with explanation.
Author response:

We thank your helpful comment. The study subjects were women who had given live birth within the 2 years before the interview. Women who had stillbirth were not included in our study. The actual number of study subjects was 3,768 but we applied sampling weights and it was changed to 3,448. We have revised the manuscript and Figure 1 accordingly.

3-5. Among 3768, 1156 were excluded due to data incompleteness; about 30.7% of the women who had given birth within 2 years were excluded. It is doubtful about the data quality in LSIS III. To prove the data quality, do you have any more justification for this, apart from the home deliveries that you have explained in the limitations?
Author response:

Thank you for this important and constructive comment. Of 3,448 women (weighted) had given live birth in the past 2 years, 2,612 (weighted) were included and 836 (weighted) were excluded. We conducted a sensitivity analysis comparing women included in the final analysis with those excluded due to missing data on key outcome variables. The results of this analysis are presented in a new table (S1 Table). Statistically significant differences were observed between the two groups across most socio-demographic and obstetric characteristics. Excluded women were more likely to have no or an elementary education, belong to ethnic minority groups as Mon-Khmer, reside in rural areas, be classified as low/middle wealth index, be married at age 19 years or younger, and have no health insurance. Nearly all excluded women delivered at home.

Lines 389-398: In response, we have revised the Limitations section to clearly state that the findings primarily reflect patterns of maternal healthcare utilization among women who delivered in health facilities and may not be generalizable to home deliveries as follows, “First, 836 of the 3,448 women who had given live birth within two years before the survey were excluded because of missing data on key variables. The excluded women differed systematically from those included, being predominantly home births and more likely to have low socioeconomic status, including having lower educational attainment, belonging to ethnic minority groups, residing in rural areas, lower/middle wealth index, being married at age 19 years or younger, and having no health insurance (S1 Table). As these characteristics are associated with lower utilization of maternal healthcare services, this exclusion may have resulted in an overestimation of the completion of the continuum-of-care. Consequently, the findings primarily reflect maternal healthcare utilization among facility-based births and may not be fully generalizable to women who had home delivery. For contextual reference, if all excluded women were classified as not completing the continuum-of-care, the overall completion rate would be 2.5% (86/3,448).”

3-6. Results: Is there any data disaggregated by duration since childbirth – within 6 months, 6-12 months, 12-18 months, and 18-24 months? It is interesting to know the differences within these groups.
Author response:

Thank you for this insightful comment. While LSIS III includes information on the timing of the most recent childbirth, the present study was designed to assess overall completion of the maternal continuum-of-care within the recommended postpartum period, rather than to examine differences by time since childbirth. Further disaggregation by shorter postpartum intervals would substantially reduce the sample size in each subgroup, potentially limiting statistical power and interpretability. We therefore focused on a binary definition of PNC within six weeks after delivery, in line with WHO recommendations. We agree that differences by time since childbirth would be an interesting topic and should be explored in future studies.

3-7. CoC coverage – from the service delivery point of view, level or rank of health staff – which may have different coverage or proportion in drop-offs within healthcare providers, so that we can know which provider should be built capacity in priorities. As the coverage is very low, we need to explore the service provider’s site as well.

Author response:

Lines 372-386: Thank you for this important comment. We agree that examining the continuum-of-care from a service delivery perspective, including the level of health facilities and health care providers, is crucial for identifying priority areas for capacity building. However, LSIS III does not provide detailed information on the cadre or rank of health staff providing maternal health services, nor does it allow differentiation of PNC by specific provider type or facility level. Therefore, an analysis of drop-offs along the continuum by provider level was beyond the scope of the present study. Nevertheless, based on the observed low coverage of PNC, especially after discharge, and existing evidence in Lao PDR, we have added a paragraph in the Discussion section to highlight potential service delivery-related barriers, including short postpartum stays at health facilities, limited counseling before discharge, and cultural practices affecting care-seeking, particularly among Hmong-Mien women. We also discuss the potential role of healthcare providers and village health volunteers in strengthening PNC uptake, especially in rural areas as follows, “To improve coverage of the maternal continuum-of-care, particularly PNC after discharge, village health volunteers may play a critical role in Lao PDR. Given the short duration of postpartum stays at health facilities and the low uptake of facility-based PNC, strengthening community-based follow-up is essential. Village health volunteers could support PNC utilization by providing targeted health education to women, husband, and senior families on the importance of PNC, identifying postpartum women in the community, and encouraging timely attendance at health facilities within the recommended postpartum period [51]. In addition, village health volunteers could assist in monitoring whether women attend scheduled postnatal visits and facilitate referrals when needed, particularly in rural and remote areas [52]. Close collaboration between healthcare providers and village health volunteers, including clear communication of PNC schedules at the time of discharge and information sharing between facilities and communities, may help reduce drop-offs along the continuum-of-care. Such community-based strategies may be especially important for ethnic minority groups, including Hmong–Mien women, among whom cultural practices and beliefs may influence care-seeking behaviors.”

3-8. Authors may provide the proportion of drop-offs across the CoC: ANC – SBA delivery – PNC, and compare with those in LSIS if possible.
Author response:

Lines 227-231: We have revised the Results section and explained decreasing the proportions as follows, “Overall, 82.2% (n = 2,147) received both ≥ 4 ANC visits and SBA-attended delivery. Only 3.3% (n = 86) completed the maternal continuum-of-care by receiving ≥ 4 ANC visits, SBA-attended delivery, and at least one PNC visit after discharge. The coverage declined markedly across the continuum, with the largest drop observed between SBA-attended delivery and PNC.”

3-9. Tables 1 and 2 – why authors put column percentages? The variable of interest is whether CoC is complete or not; we can compare across the categories of independent variables by row percentages, which is more informative. Or, why do you choose column percentage? Please give justification. The table data explanation also emphasizes only frequency within variables, no description related to CoC Yes or No, with the p-value (Chi-Square Test results in tables), whether significant or not. It is better to describe them here.
Author response:

Thank you for this important comment. Tables 1 and 2 were intended to describe the baseline characteristics of the study population by the continuum-of-care completion status using column percentages, which allows comparison of the profiles of women with and without complete the continuum-of-care. The association between women’s characteristics and the continuum-of-care completion (i.e., which groups had higher or lower the continuum-of-care completion) was formally assessed using univariate logistic regression analysis, as presented in Table 3. Therefore, we consider Table 3 as the primary table addressing this research question, while Tables 1 and 2 serve as descriptive summaries of the study population.

3-10. Conclusion: Do you have any recommendations for healthcare providers’ sites?

Author response:

Lines 420-429: Thank you for your helpful comment. We have revised the Conclusion to explicitly separate recommendations from the healthcare provider’s perspective and recommendations at the system level as follows, “From the healthcare providers’ perspective, strengthening post-discharge PNC is critical. Healthcare providers at health facilities should ensure that women receive standardized maternal health checks and clear counseling on the importance and timing of PNC before discharge and that follow-up PNC visits are scheduled at the time of delivery. Strengthening links between health facilities and village health volunteers is also essential. At the system level, efforts by the Lao government to promote integrated maternal health services, reduce financial barriers, and address gender norms and inequities remain critical. In addition, strengthening MNCH services that are responsive to the specific cultural practices and beliefs of the Hmong-Mien community may improve both access to and completion of the continuum-of-care.”

**Reviewer #4**: The manuscript addresses an important public health issue by examining the coverage of the maternal continuum of care (CoC) in the Lao People’s Democratic Republic (Lao PDR). The use of a population-based, cross-sectional study design is appropriate for estimating coverage proportion and identifying determinant factors. It could contribute valuable insights for informing national maternal health strategies and policy prioritization. Here are specific recommendations for the authors to consider:
1. In the introduction section, revise the 1st and 2nd paragraphs to be a logical flow of the problem statement. For example, Line No. 58-59, sudden transition to the SDG targets without joint information makes it abrupt and confused for the readers. Structural improvement is needed in these phrases.
Author response:

Lines 42-68: We have revised the first and second paragraphs of the Introduction to improve the logical flow of the problem statement as follows, “The continuum-of-care for maternal, newborn, and child health (MNCH) refers to the integrated and uninterrupted provision of services throughout pregnancy, childbirth, and the postpartum period, including antenatal care (ANC), intrapartum care, and postnatal care (PNC) [1-3]. An integrated system to deliver the continuum-of-care for MNCH is essential to reduce morbidity and mortality among mothers, newborns, and children [1]. The World Health Organization (WHO) recommends at least eight ANC visits to promote women’s health during pregnancy [1, 4]. For intrapartum care, deliveries assisted by skilled birth attendants (SBA) at health facilities are recommended to prevent maternal deaths [5, 6], and timely PNC visits after discharge from health facilities to detect and manage postpartum complications [1, 7]. A systematic review showed that completing the continuum-of-care significantly reduces neonatal mortality, decreasing the risk of neonatal death by 16% [2].

Although substantial progress has been made in reducing maternal and child mortality globally, many low- and middle-income countries (LMICs) continue to experience preventable maternal and child deaths, and the pace of improvement remains insufficient [8-10]. Persistent gaps in access to and continuity of MNCH services, particularly during the postpartum period, contribute to these outcomes. In this context, improving the coverage and continuity of essential MNCH services has become a global priority. Reflecting this urgency, the Sustainable Development Goals (SDGs) set targets to reduce the global maternal mortality ratio (MMR) to fewer than 70 per 100,000 live births and the under-five mortality rate to fewer than 25 per 1,000 live births by 2030 [8-10].

Globally, the coverage of the continuum-of-care varies substantially across countries, particularly in relation to their level of socioeconomic development. In high-income countries, coverage across all three stages of the continuum-of-care is high, approaching 100%. In LMICs, coverage remains low, especially in sub-Saharan Africa and parts of South Asia [11-19]. A systematic review analyzing data from 17 sub-Saharan African countries found that the continuum-of-care for MNCH declined at each successive stage, with most women receiving ANC and intrapartum care, but not PNC [20].”

4-2. In the methods section, while describing the socio-demographic factors (Pg No. 6, Line no. 131-132), it’s better to recheck the wealth index quintiles (low, middle, upper, and high). The first thing is that if the authors express wealth index in quintiles, it should be 5 groups. If only four groups, it should be quantile. Please make it clear since it could change the final results. Also, check the original dataset and confirm how they mention the wealth status in their report.
Author response:

Lines 137-139: We apologize for our mistake of the wealth index categories. In LSIS III, the wealth index quintiles included poorest, second, middle, fourth, and richest. In our study, we categorized into “low/middle” (including poorest, second, and middle) and “high” (including fourth and richest). We have revised the Methods section as follow, “Wealth index quintiles (poorest, second, middle, fourth, and richest) were grouped into two categories: “low/middle (poorest, second, and middle)” and “high (fourth and richest)”.”

4-3. In the methods section, while describing the obstetric factors (Pg No. 7, Line no. 151-155), delivery assistant includes “other” category referring to traditional birth attendants and village health volunteers. However, in the place of delivery, the categories are “hospital,” “health center,” or “private clinic” only. It makes a little bit confused whether home delivery is included or not. Please recheck the data and add this group if relevant.
Author response:

Thank you for this important comment. We would like to clarify that the analytical sample was restricted to women who reported facility-based delivery only. Women with missing information on ANC visits, delivery assistant, or PNC were excluded from the analysis; as a result, all women who delivered at home were excluded from the study population. Although delivery took place in a health facility (hospital, health center, or private clinic), a small number of women reported the delivery assistant as “traditional birth attendant” or “village health volunteer.” This reflects self-reported responses in the LSIS III dataset. As this study is a secondary data analysis, we used the delivery assistant variable as recorded in the original dataset without reclassification. We acknowledge that, in practice, deliveries in health facilities are typically attended by SBA, and this discrepancy likely reflects reporting or classification issues in the survey rather than actual home deliveries.

Lines 115-119: To avoid confusion, we have revised the Methods section as follow, “The analytical sample was restricted to women who reported facility-based delivery, defined as delivery at a hospital, health center, or private clinic. Women with missing information on ANC visits, delivery assistant, or PNC were excluded from the analysis; consequently, women who delivered at home were not included in the study population.”

4-4. In the methods section, while describing the maternal CoC (Pg No. 8, Line no. 162), the CoC was defined as receiving ANC at least four times. Meanwhile. the WHO's current recommendation is eight ANC contacts. Here, the authors should clearly articulate the rationale for this specific operational definition in the context of Lao PDR's national guidelines, if they differ from the WHO standard. Moreover, in Line No. 166-167, it’s better to mention types of healthcare provider for standard postpartum care in the operational definition.
Author response:

Lines 181-183: Thank you for your comment. We acknowledge that the definition of maternal continuum-of-care used in this study is less strict than current WHO recommendations, which include at least eight antenatal contacts and PNC at specific time intervals. Due to limitations in available LSIS III variables and the extremely low uptake of PNC, applying WHO-recommended thresholds was not feasible in this study. Regarding the number of ANC visits, we have added a sentence to the Methods section as follows, “Although the WHO recommends ≥ 8 ANC contacts, this was not fully implemented in Lao PDR during the LSIS III period, and the national strategy still uses ≥ 4 ANC visits as an indicator.”

Lines 185-187: We have added a sentence to explain recommended PNC providers as follows, “Although the continuum-of-care in this study did not distinguish between the types of providers, in Lao PDR, PNC is generally recommended to be provided by qualified healthcare providers, such as doctors, midwives, or nurses.”

Lines 399-403: We have revised the Limitations section to clearly state that the use of lower thresholds may overestimate continuum-of-care completion and restrict comparability with studies using WHO-recommended definitions as follows, “Second, the definition of maternal continuum-of-care in this study is less stringent than current WHO recommendations regarding the number of ANC visits and was necessitated by data constraints and very low PNC uptake, which may limit comparability with other studies. Completion rate of the continuum-of-care when using at least ANC 8 visits was 1.7% (45/2,612).”

4-5. In the methods section, while statistical analysis (Pg No. 8), it would be beneficial to add a diagnostic check for multicollinearity among the predictor variables used in the final adjusted model. Specifically, please calculate and report the Variance Inflation Factor (VIF) for each variable in the multivariable logistic regression. This step is crucial for confirming that the standard errors and p-values are not disproportionately inflated due to linear dependencies among the covariates
Author response:

Thank you for this valuable suggestion. We assessed multicollinearity among all variables included in the adjusted logistic regression model using the Variance Inflation Factor (VIF). All VIF values were below 5, indicating that multicollinearity was not a concern. This information has been added to the Statistical analysis section of the Methods. Please see our response to Comment No. 1-3.

4-6. In the results section, (Pg No. 9, Line no. 195-199), most information is repeatedly described, better to remove them and rephrase to become more concise results.
Author response:

Lines 224-235: Thank you for this helpful comment. We agree that the initial version of the Results section contained redundant information overlapping with the Methods. We have revised the Results section to remove repeated descriptions of participant selection and exclusion criteria. The Results section has been rephrased to focus more concisely on the coverage of each maternal care component and the completion of the continuum-of-care as follows, “A total of 2,612 women were included in the final analysis (Fig 1) after excluding 836 who did not respond to questions about the number of ANC and PNC visits. Among these women, 83.1% (n = 2,170) received at least four ANC visits, 98.6% (n = 2,576) had delivery attended by an SBA, and 3.8% (n = 98) received at least one PNC visit after discharge. Overall, 82.2% (n = 2,147) received both ≥ 4 ANC visits and SBA-attended delivery. Only 3.3% (n = 86) completed the maternal continuum-of-care by receiving ≥ 4 ANC visits, SBA-attended delivery, and at least one PNC visit after discharge. The coverage declined markedly across the continuum, with the largest drop observed between SBA-attended delivery and PNC. Women who delivered at home (n = 736) were excluded from the analytical sample; therefore, all 2,612 women included in the analysis had facility-based deliveries. Characteristics of included and excluded women, including those with home deliveries, were compared and are presented in S1 Table.”

4-7. Please re-test the statistical analysis and rearrange the tables if the authors need to revise the variables “wealth index quintiles” and “place of delivery”.
Author response:

Thank you for this comment. We have carefully rechecked the original LSIS III dataset and confirmed that the wealth index was originally constructed as quintiles (poorest, second, middle, fourth, and richest). As noted above, we corrected the description and grouping of the wealth index in the Methods section and updated the Results and tables accordingly. Regarding place of delivery, the analytical sample was restricted to women with facility-based deliveries (hospital, health center, or private clinic) from the outset, and this definition was not revised. Therefore, no changes to the variable coding or analytical sample were required. After confirming these points, we verified that the statistical analyses and key findings were unchanged. Accordingly, re-testing the analyses or further rearrangement of the tables was not necessary.

4-8. The discussion and conclusion parts are well constructed with relevant contrast.

Author response:

Thank you for this positive comment. We note that revisions were made to the Discussion and Conclusion sections in response to comments from other reviewers; however, the overall structure and key messages remain unchanged.

**Reviewer #5**: This manuscript addresses an important maternal health issue and provides valuable insights into antenatal care (ANC) and postnatal care (PNC) utilization among women in the Lao People’s Democratic Republic. The topic is highly relevant to public health programs, particularly in low- and middle-income countries where barriers to maternal health services remain significant. The study objectives are clearly stated, and the use of population data provides useful implications for service improvement. However, there are some areas that require additional clarification and refinement to enhance the accuracy, interpretability, and practical value of the findings.

5-1. Line 134, “never married”: In many Asian cultural contexts, women typically pursue pregnancy within marriage. Therefore, the definition of the variable “never married” requires clarification. If “never married” refers to women who have never been in a marital union, they would logically fall under the exclusion criteria. Alternatively, if “never married” includes single mothers or women who became pregnant outside of marriage, this should be explicitly stated.

I would like to recommend that the authors clarify the operational definition of “never married” in the study.

Author response:

Thank you for this important comment. In LSIS III, marital status is assessed using two separate questions: (1) current marital/union status (“currently married,” “living with a man,” or “not in union”) and (2) history of marital/union experience (“formerly married,” “formerly lived with a man,” or “no”). In our analysis, women were categorized as “never married” if they reported “not in union” for current marital status and “no” for ever having been married or lived with a man. Importantly, LSIS III allows for the inclusion of women who have experienced pregnancy and childbirth outside of marriage. Therefore, women classified as “never married” in this study may include single mothers who gave birth outside a marital union. Our study population was defined based on reproductive history (women aged 15–49 years who had a live birth within the two years preceding the survey), not marital status. As such, women categorized as “never married” were not excluded if they met the inclusion criteria.

Lines 139-143: We have revised the Methods section to clarify the operational definition of marital status as follows, “Marital status was categorized as married (formally married or living with a partner) and not married (divorced, widowed, or never married). “Never married” refers to women who reported neither current nor previous marital or cohabiting unions; this category may include women who experienced childbirth outside of marriage.”

5-2. Line 229, ANC Provider “Other” and Place of Delivery: In Line 150, the “Other” category for ANC providers includes traditional birth attendants, village health volunteers, and relatives. Women who choose providers in this category are often more likely to give birth at home or in locations outside formal health facilities.

In Table 2, the authors report that 33 (1.3%) of women delivered with providers categorized as “Other.” However, there appears to be no corresponding category defined in Lines 153–154 or clearly reflected in the “Place of Delivery” in Table 2.

I recommend that the authors clarify the definition of the “Other” ANC provider category and verify these data across the manuscript.

Author response:

In LSIS III, ANC providers are categorized as doctor, nurse/midwife, traditional birth attendant, village health volunteer, and “other.” Birth assistants are categorized separately and include doctor, nurse/midwife, traditional birth attendant, village health volunteer, relatives/friends, and “other.” In our analytical sample (n = 2,612), all women reported facility-based delivery (hospital, health center, or private clinic), as women with missing information on ANC visits, delivery assistant, or PNC were excluded, which resulted in the exclusion of all women who delivered at home. Nevertheless, a small number of women reported their ANC provider or delivery assistant as “other,” despite reporting delivery at a health facility. Specifically, 2 women reported “other” as their ANC provider and 4 women reported “other” as their delivery assistant. Detailed information on the specific type of provider included in the “other” category is not available in the LSIS III dataset. As this study is a secondary data analysis, we used the provider categories as recorded in the original dataset without reclassification. We acknowledge that, in practice, deliveries in health facilities are typically attended by SBA, and this discrepancy likely reflects reporting or classification limitations in the survey rather than actual home deliveries. To avoid confusion, we have clarified the operational definitions of ANC providers and delivery assistants in the Methods section and revised the footnotes of Tables 2 and 3 accordingly.

Lines 159-167: We have also revised the Methods section as follows, “The ANC provider was defined as the person who primarily provided ANC to the woman—categorized as “doctor,” “nurse/midwife,” or “other” (including traditional birth attendants, village health volunteers, and other). Mode of delivery was categorized as either “vaginal delivery” or “cesarean section.” Delivery assistant referred to the individual who assisted during childbirth and was grouped as “doctor,” “nurse/midwife,” or “other” (including traditional birth attendants, village health volunteers, relatives/friends, and others). The analytical sample was restricted to women who reported facility-based delivery; however, a small number of women reported “other” as the delivery assistant despite facility-based delivery.”

5-3. Lines 275-277, Family permission; Line 298, “Women who received postpartum checks.”

I strongly support the authors’ point, which aligns with the socio-cultural context in many Asian settings. In these societies, decision-making regarding antenatal and postnatal care is often influenced by husbands and elder family members.

Therefore, I would like to recommend that the authors consider adding wording that highlights the importance of engaging husbands and senior family members in promoting ANC and PNC utilization.

Author response:

Lines 310-315: Thank you for this insightful and supportive comment. We fully agree that, in many Asian socio-cultural contexts, decision-making regarding maternal healthcare utilization is strongly influenced by husbands and senior family members. In response to this suggestion, we have revised the Discussion section to explicitly highlight the importance of engaging husbands and elder family members in promoting both ANC and PNC utilization. Specifically, we have added wording emphasizing that interventions and health education efforts should target not only women themselves but also key family decision-makers to improve maternal health service uptake as follow, “Additionally, some women may require family permission to leave the house or may not receive adequate information about PNC during ANC visits or at the time of delivery [36]. To improve PNC coverage, family engagement in maternal health should be promoted, accurate information regarding the importance of PNC should be provided, and implementation of standard discharge protocols, including the scheduling of PNC visits, should be strengthened [3, 7, 36, 39].”

5-4. Lines 300-301, Such information and counseling could have facilitated subsequent utilization of PNC services.

From a practical implementation perspective, I would like to suggest that the authors consider adding the local language(s) in this point to be better understanding, to enhance comprehension, ensure cultural relevance, and improve the effectiveness of PNC promotion efforts.

Author response:

Lines 344-347: Thank you for this valuable and practical suggestion. We agree that the use of local language(s) is important for improving comprehension and cultural relevance of maternal health counseling. Accordingly, we have revised the sentence as follows, “Such information and counseling could have facilitated subsequent utilization of PNC services, particularly when they were provided in local languages that women and their families can easily understand, thereby enhancing comprehension and cultural relevance.”

5-5. Lines 310-322, Different strategy

The authors correctly highlight key elements such as improving knowledge and implementing culturally appropriate strategies. Previous studies have demonstrated that context-specific approaches, including home visits in geographically challenging or mountainous regions, can be particularly effective. Additionally, the involvement of ethnic community leaders has been shown to play an important role in promoting ANC and PNC uptake within their communities.

From a programmatic and practical standpoint, I would like to recommend that the authors consider elaborating on geographically and culturally tailored interventions such as home-based outreach and collaboration with ethnic community leaders to strengthen the applicability of their recommendations.

Author response:

Lines 368-371: Thank you for this constructive and practical suggestion. We agree that context-specific and culturally tailored interventions are important for improving ANC and PNC utilization among Hmong-Mien women. We have added a sentence to the paragraph of the Discussion section as follows, “In addition, geographically and culturally tailored interventions, such as home-based outreach or home visits in remote or mountainous areas, and collaboration with ethnic community leaders may be effective in promoting ANC and PNC utilization by addressing both geographic barriers and culturally embedded decision-making processes.”

**Reviewer #6**: This study is valuable and may stimulate government officials to improve maternal, newborn, and child health in developing countries worldwide. Factors influencing the low completion of the maternal continuum of care can be potentially applied to improve maternal, newborn, and child health in the study area.

Author response:

Thank you very much for this positive and encouraging comment. We appreciate the reviewer’s recognition of the potential policy and programmatic relevance of our findings. We hope that the identification of factors associated with low completion of the maternal continuum-of-care will help inform strategies to strengthen maternal, newborn, and child health services in similar low- and middle-income settings.

**Reviewer #7**:

The study is well designed, and the data support the authors’ conclusions. The authors used the right statistical methods and analyzed the data carefully. The data used in the study are accessible to the public through the MICS website. The paper is easy to understand and written in acceptable English. The study is important for improving maternal health in Lao PDR. The authors mention the study’s limitations and give useful recommendations. They should explain how to address cultural barriers and how their findings can guide policy. Overall, the paper is clear and useful.

7-1. The manuscript identifies that Hmong-Mien women are significantly less likely to complete the continuum of care, mentioning cultural practices and language barriers. However, the discussion remains general and does not provide actionable recommendations or detailed strategies to address these barriers.
Author response:

Lines 368-371: Thank you for this constructive and practical suggestion. We agree that context-specific and culturally tailored interventions are important for improving ANC and PNC utilization among Hmong-Mien women. We have added a sentence to the paragraph of the Discussion section as follows, “In addition, geographically and culturally tailored interventions, such as home-based outreach or home visits in remote or mountainous areas, and collaboration with ethnic community leaders may be effective in promoting ANC and PNC utilization by addressing both geographic barriers and culturally embedded decision-making processes.”

7-2. While the manuscript recommends strengthening health insurance and reducing financial barriers, it does not offer concrete examples of how these can be implemented or scaled up in Lao PDR.
Author response:

Thank you for this constructive comment. We agree that providing more concrete examples would strengthen the practical relevance of our recommendations. We have revised the Discussion section to include specific, context-appropriate examples for Lao PDR, such as expanding enrollment and retention in the national health insurance scheme, introducing transportation vouchers or conditional cash transfers for pregnant and postpartum women in rural areas, and strengthening community-based referral and transport support. These additions aim to clarify how financial barriers to completing the continuum-of-care could be addressed in practice.

Lines 336-341: We have revised the paragraph of the Discussion section as follows, “Concrete measures may include strengthening enrollment and continuity in the national health insurance scheme, providing transportation vouchers or conditional cash transfers for pregnant and postpartum women in rural or remote areas, and supporting community-based referral and transport mechanisms to facilitate timely access to health facilities. These approaches could help mitigate indirect costs and improve equitable access to MNCH services.”

7-3. The paper highlights low postnatal care (PNC) utilization and the importance of family engagement but does not cite or describe successful programs from other contexts that could serve as models.
Author response:

Lines 372-378: Thank you for this helpful suggestion. We agree that providing examples of successful programs can strengthen the practical relevance of our discussion. In the Discussion section, we have added a paragraph of recommendations informed by both our study’s findings and evidence from other low- and middle-income country settings, including community-based approaches (home visits by village health volunteers and the involvement of husbands and senior family members) as follows, “To improve coverage of the maternal continuum-of-care, particularly PNC after discharge, village health volunteers may play a critical role in Lao PDR. Given the short duration of postpartum stays at health facilities and the low uptake of facility-based PNC, strengthening community-based follow-up is essential. Village health volunteers could support PNC utilization by providing targeted health education to women, husband, and senior families on the importance of PNC, identifying postpartum women in the community, and encouraging timely attendance at health facilities within the recommended postpartum period [51].”

7-4. The authors note that women who delivered at home were excluded due to missing data but do not discuss how this exclusion may bias the results or affect generalizability.
Author response:

Lines 387-398: We have revised the Limitations section to clarify potential bias and generalizability issues as follows as follows, “First, 836 of the 3,448 women who had given live birth within two years before the survey were excluded because of missing data on key variables. The excluded women differed systematically from those included, being predominantly home births and more likely to have low socioeconomic status, including having lower educational attainment, belonging to ethnic minority groups, residing in rural areas, lower/middle wealth index, being married at age 19 years or younger, and having no health insurance (S1 Table). As these characteristics are associated with lower utilization of maternal healthcare services, this exclusion may have resulted in an overestimation of the completion of the continuum-of-care. Consequently, the findings primarily reflect maternal healthcare utilization among facility-based births and may not be fully generalizable to women who had home delivery. For contextual reference, if all excluded women were classified as not completing the continuum-of-care, the overall completion rate would be 2.5% (86/3,448).”

7-5. While the manuscript is generally clear, it will be better after minor revisions, such as lengthy explanations and a listing of variables in the methods section.
Author response:

Thank you for this constructive comment. We have carefully revised the manuscript to improve clarity and conciseness. Specifically, we shortened lengthy explanations and streamlined the Methods section by reducing repetitive descriptions and organizing the list of variables more clearly. These revisions were made to enhance readability while maintaining methodological transparency.

7-6. Since statistically significant results are not visually distinguished from non-significant ones in Tables 1, 2, and 3, it needs to consider bolding or marking statistically significant p-values or using symbols (e.g., * for p < 0.05).
Author response:

In response to the reviewer’s comment, we revised Tables 1–3 so that statistically significant p-values (p < 0.05) are highlighted in bold, while non-significant p-values remain in the standard format, in accordance with common reporting practices.

7-7. It should expand the caption to clarify what Table 3 shows (e.g., “Table 3. Bivariate and multiple logistic regression analysis of factors associated with completion of the maternal continuum-of-care among the participants (N=2,612)”).

Author response:

Thank you for this helpful suggestion. We have revised the title of Table 3 to more clearly describe the content of the analysis as follows, “Bivariate and multiple logistic regression analysis on factors associated with completion of the maternal continuum-of-care among the participants (N=2,612).”

**Reviewer #8**:

8-1. How exactly was PNC defined in LSIS III and in your analysis? Does LSIS capture immediate postnatal checks before discharge? If yes, please include it or justify exclusion.
Author response:

Lines 176-185: Thank you for your comment. In LSIS III, PNC was defined as any maternal health check received after delivery, including visits at a health facility or at home. While LSIS III captures immediate postnatal checks before discharge, these were not included in our definition of PNC in this study. In our analysis, PNC was defined as at least one visit occurring after discharge from the health facility up to six weeks postpartum. We excluded the routine discharge checks from the PNC variable because nearly all women delivering at health facilities receive these checks, and therefore they do not contribute to variation in continuum-of-care completion. By focusing on PNC after discharge, our definition better reflects gaps in continuity of maternal care and aligns with the study objective of assessing the maternal continuum-of-care. We have revised the section of “Maternal continuum-of-care” in the Methods section as follows, “In this study, the continuum-of-care was defined as receiving ANC at least four times, delivery attended by an SBA; including doctors, midwives, and nurses, and at least one PNC visit after discharge from a health facility within six weeks postpartum, as routine facility-based checks prior to discharge are nearly universal and do not capture continued care-seeking after delivery. Participants who received all three components were considered to have completed the continuum-of-care. Although the WHO recommends ≥ 8 ANC contacts, this was not fully implemented in Lao PDR during the LSIS III period, and the national strategy still uses ≥ 4 ANC visits as an indicator. Due to the limited number of women who received PNC, having at least one PNC visit within six weeks after delivery was deemed sufficient to meet the PNC criterion.”

8-2. Did you account for survey strata and cluster/PSU in SPSS (Complex Samples)? Please specify the variables and procedures used.
Author response:

While we used the survey weights provided in LSIS III, we did not account for stratification or clustering in SPSS due to software limitations. We acknowledge that this may slightly underestimate standard errors, but the main effect estimates are unlikely to be materially affected.

8-3. Please provide a weighted comparison of included vs excluded women (n=2,612 vs n=1,156 excluded) to assess selection bias.
Author response:

We have made a new table “S1 Table” to show the comparison of characteristics between “included women” and “excluded women.” In lines 387-398 of the Discussion section, we have revised a paragraph about limitations as follows, “First, 836 of the 3,448 women who had given live birth within two years before the survey were excluded because of missing data on key variables. The excluded women differed systematically from those included, being predominantly home births and more likely to have low socioeconomic status, including having lower educational attainment, belonging to ethnic minority groups, residing in rural areas, lower/middle wealth index, being married at age 19 years or younger, and having no health insurance (S1 Table). As these characteristics are associated with lower utilization of maternal healthcare services, this exclusion may have resulted in an overestimation of the completion of the continuum-of-care. Consequently, the findings primarily reflect maternal healthcare utilization among facility-based births and may not be fully generalizable to women who had home delivery. For contextual reference, if all excluded women were classified as not completing the continuum-of-care, the overall completion rate would be 2.5% (86/3,448).”

8-4. With only 86 events, please justify the multivariable model complexity and report events-per-variable; consider a parsimonious model or penalized methods.
Author response:

We thank the reviewer for raising important concerns regarding potential overfitting and model specification.

We acknowledge that the multivariable model includes 11 variables with 86 outcome events, which may raise concerns about the events-per-variable ratio. However, all variables were selected a priori based on established literature and theoretical relevance to control for potential confounding, rather than through data-driven selection procedures.

To address concerns about model stability, we assessed multicollinearity among all covariates using VIFs. All VIF values (1.009-1.743) were below commonly accepted thresholds (< 2.0), indicating no evidence of problematic multicollinearity. In addition, we conducted sensitivity analyses using alternative, more parsimonious models including only key covariates. The direction and magnitude of the main effect estimates were consistent with those of the primary model, supporting the robustness of our findings.

Lines 199-205: We have added sentences in the Methods section as follows, “Selection of variables for the multivariable model was determined a priori based on prior literature and theoretical relevance, with the aim of adequately controlling for potential confounding factors. To assess potential multicollinearity among covariates, variance inflation factors (VIFs) were calculated for all variables included in the multivariable model. All VIF values were below commonly accepted thresholds, indicating no evidence of problematic multicollinearity. Model diagnostics were performed to evaluate the adequacy of the final model and model fit was assessed using the Hosmer–Lemeshow goodness-of-fit test.”

Lines 282-287: We have added sentences in the Results section as follows, “Sensitivity analyses were conducted to assess the robustness of the multivariable model. Alternative models, including only key covariates selected based on theoretical importance, were examined. The direction and magnitude of the main effect estimates were consistent with those observed in the primary model, indicating that the results were not materially affected by the number of covariates included.”

We believe these additional analyses and clarifications adequately address the reviewer’s concerns regarding overfitting and model robustness.

8-5. Please correct Table 1 education percentage (869 cannot be 3.33% of 2,612) and re-check all tables.
Author response:

Table 1: We are very sorry to write the wrong percentage and we have revised “3.33%” to “33.3%.”

8-6. Please provide the ethics approval number for LSIS III (or explain why unavailable).

Author response:

Thank you for this comment. The present study is a secondary analysis of data from the LSIS III, which is part of the UNICEF-supported Multiple Indicator Cluster Surveys (MICS) program. LSIS III received ethical approval from the National Ethics Committee for Health Research of the Lao People’s Democratic Republic, as described in the LSIS III final report. However, the ethics approval number was not reported in the publicly available documentation. As this study involved secondary analysis of anonymized, publicly available data, additional ethical approval was not required.

**Reviewer #9**:

9-1. Regarding the outcome measures, line number 162-167 say “the continuum-of-care was defined as receiving ANC at least four times, delivery attended by an SBA; including doctors, midwives, and nurses, and at least one PNC visit after discharge from a health facility within six weeks postpartum. Participants who received all three components were considered to have completed the continuum-of-care. Due to the limited number of women who received PNC, having at least one PNC visit within six weeks after delivery was deemed sufficient to meet the PNC criterion.”

So, among 2617 study participants, 2170 got more than 4 ANC visits, 2147 got SBA delivery but only 86 got PNC. Given that nearly all women delivered in health facilities (n = 2,612), including 252 cesarean sections, it is unclear whether women who had SBA-assisted institutional deliveries did not receive any postnatal assessment, including within 24 hours of delivery. Postnatal care was operationalized as a separate maternal health check occurring after discharge from the delivery facility. This strongly suggests that routine postpartum checks before discharge were not classified as PNC.
This results low prevalence of complete continuum of care (3.3%) largely due to the restricted operational definition of postnatal care rather than a true absence of services, which may affect the outcome estimates and may create a highly imbalanced outcome distribution which could affect the precision and stability of multivariable regression estimates.
As a result, the low outcome prevalence (3.3%) leads to a highly imbalanced outcome distribution and a relatively small number of events despite the large study population. Under these conditions, conventional logistic regression would be prone to small-sample bias, overfitting of predictors, and an increased risk of type I error. Reducing the number of predictors or applying penalized logistic regression methods is therefore recommended to minimize overfitting and prevent inflation of odds ratio estimates.

Author response:

We agree that postnatal assessments prior to discharge are routinely provided for women delivering in health facilities, and therefore acknowledge that most women in our study likely received some form of immediate postpartum care before discharge. However, the primary objective of this study was to evaluate continuity of maternal care beyond facility-based delivery, with a particular focus on post-discharge PNC, which is widely recognized as critical for preventing maternal morbidity and mortality. For this reason, we deliberately operationalized PNC as at least one maternal health check occurring within six weeks after discharge, rather than including routine postpartum assessments conducted prior to discharge.

We acknowledge that this restrictive definition resulted in a low prevalence of complete continuum-of-care (3.3%), leading to a highly imbalanced outcome distribution and a relatively small number of events. To address concerns regarding potential overfitting and small-sample bias in multivariable logistic regression, we limited the number of predictors included in the final model based on prior literature and theoretical relevance (please see our response to comment No. 1-3). In addition, we carefully interpreted adjusted odds ratios with caution and emphasized consistency of direction and magnitude rather than statistical significance alone.

Furthermore, we have added this issue explicitly to the Limitations section, noting that the low outcome prevalence may affect the precision and stability of the regression estimates. Despite these limitations, we believe that the extremely low uptake of post-discharge PNC observed in this study represents a substantive gap in maternal health service utilization in Lao PDR rather than merely an artifact of outcome definition. Highlighting this gap is of high public health relevance and underscores the need to strengthen post-discharge follow-up care.
