# Supplementary material for: The coverage of maternal continuum-of-care and associated factors in the Lao People’s Democratic Republic: A population-based cross-sectional study
Source: PLoS One. 2026 Mar 25;21(3):e0345660. doi: 10.1371/journal.pone.0345660 (PMC13016336; doi:10.1371/journal.pone.0345660)
Supplement: Attachment — Submitted filename: Response_to_reviewers_auresp_2.docx [file pone.0345660.s006.docx]

**Response to the editor’s and reviewers’ comments**

We would like to thank the editor and reviewers for reviewing our manuscript. We have revised the manuscript according to your comments, which were very helpful. The revised manuscript has been proofread by a native English speaker. The revisions have been completed and our responses are as follows.

**Journal requirements:**

1. If the reviewer comments include a recommendation to cite specific previously published works, please review and evaluate these publications to determine whether they are relevant and should be cited. There is no requirement to cite these works unless the editor has indicated otherwise.

Author response:

There was no comment from the reviewers that includes a recommendation to cite specific previously published works.

**Additional Editor Comments:**

1. Line 107: Please change to Study participants
Author response:

Lines 107: We have revised “Study subjects” to “Study participants.”

2. Line 123: Proof read the line
Author response:

Line 123: Thank you for the suggestion. We have revised to “Of the 22,126 women aged 15–49 years who participated in LSIS III (unweighted n = 22,126).”

3. Line 129: I suggest to change to- Socio-demographic characteristics

Author response:

Line 129: Thank you for the suggestion. We have revised “Socio-demographic factors” to “Socio-demographic characteristics.”

4. Line 150 and following paragraph- Tighten your language, I may suggest to include all the variables into a single table or subheading including all the variable definition.
Author response:

Lines 150-156: Thank you for the helpful suggestion. We have revised this section to improve conciseness and clarity. The definitions and categorizations of obstetric variables have been summarized in a new table (S1 Table), and the text in the Methods section has been shortened accordingly.

5. Line 223: I suggest to change to – Maternal Care service status
Author response:

Line 206: Thank you for the suggestion. The subheading has been revised from “The coverage of maternal care” to “Maternal care service status” as recommended.

6. Line 237- I suggest to change to - Socio-demographic factors of the study subjects into Socio-demographic Characteristics of the study participants
Author response:

Line 220: Thank you for the suggestion. We have revised “Socio-demographic factors of study subjects” to “Socio-demographic characteristics of study participants.”

7. Table 1. use n= ....rather than N and format table as per PLOS One standards
Author response:

Thank you for the suggestion. We have revised Tables 1 and 2 to use “n” instead of “N” and reformatted the tables according to the PLOS One table style.

8. Line 247: the title is not representing the table you are presenting. You are showing the Cross tabulation, please review similar paper and format accordingly. I shall suggest to take consultation with an experienced statistician.
Author response:

Thank you for the helpful comment. We have revised the titles of Tables 1 and 2 to better reflect the content of the tables. The new title of Table 1 is “Socio-demographic characteristics of women according to completion of the maternal continuum-of-care.” The new title of Table 2 is “Obstetric characteristics of women according to completion of the maternal continuum-of-care.” The tables present cross-tabulations of socio-demographic variables by continuum-of-care completion status.

9. Line 266: the sub-heading should be something like- Association between socio-demographic factors and completion of maternal continuum of care rather than - Logistic regression analysis on completion of the maternal 267 continuum-of-care
Author response:

Lines 250-251: Thank you for the helpful suggestion. We have revised the subheading to better reflect the content of the analysis. The new subheading is “Factors associated with completion of the maternal continuum-of-care,” because not only socio-demographic factors but also obstetric variables were included.

10. Table 3. is perfect but format for clarity and we dont need P value here. In the footnote highlight which aOR or cOR is significant based on OR
Author response:

Thank you for your valuable comment. In Table 3, P-values are presented together with crude odds ratios (cOR) and adjusted odds ratios (aOR) to provide full statistical information. Statistical significance was defined as *P* < 0.05 and this has been clearly stated in the Methods section. In addition, statistically significant *P*-values are already highlighted in bold in the table, which allows readers to easily identify significant associations. Therefore, we believe that the current format provides clear and complete statistical reporting. For these reasons, we have retained the P-values in Table 3.

11. Discussion: First paragraph overall summarize your study findings. I suggest to rewrite your whole section analytically.
Author response:

Lines 282-288: Thank you for your valuable comment. We agree that the first paragraph of the Discussion should clearly summarize the key findings of the study. Therefore, we have revised the first paragraph to more clearly present the main results, including the overall coverage of the maternal continuum-of-care, the stage at which discontinuation most commonly occurred, and the factors associated with completion of the continuum-of-care. We have revised the first paragraph as follows, “This study examined the coverage and determinants of completion of the maternal continuum-of-care in Lao PDR using nationally representative LSIS III data. The findings revealed that the overall coverage of the maternal continuum-of-care was low (3.3%). Discontinuation of care most commonly occurred at the PNC stage. Factors associated with completion of the continuum-of-care included a higher wealth index, having the last child as male, and receiving a maternal health check before discharge. In contrast, Hmong-Mien women were less likely to complete the continuum-of-care than Lao-Tai women.”

12. Your discussion is too long and loosely written, I suggest to rewrite analytically and tighten the results.
Author response:

Thank you for your valuable comments. In response to your suggestion, we have substantially revised the Discussion section to improve clarity and analytical structure. The section has been shortened and reorganized to focus more clearly on the key findings and their interpretation. Specifically, we streamlined several paragraphs to avoid repetition and tightened the discussion of policy implications.

13. Where is your strength and limitation of the study, please include these too.
Author response:

Lines 351-356: We have added a new paragraph describing the strengths of the study and combined the strengths and limitations into a dedicated subsection in the Discussion.

14. Conclusion: Rework on conclusion, your writing need to tighten. Conclude based on the information.
Author response:

Lines 380-387: Thank you for your valuable comment. We have revised the Conclusion section to make it more concise and focused on the key findings of the study. The revised conclusion summarizes the main results and highlights their implications for improving maternal continuum-of-care in Lao PDR, while removing detailed policy descriptions to improve clarity and brevity.
